# Vesicle trafficking in rice: too little is known

**DOI:** 10.3389/fpls.2023.1263966

**Published:** 2023-09-18

**Authors:** Xiaobo Zhu, Junjie Yin, Hongming Guo, Yuping Wang, Bingtian Ma

**Affiliations:** ^1^ State Key Laboratory of Crop Gene Exploration and Utilization in Southwest China, Rice Research Institute, Sichuan Agricultural University at Wenjiang, Chengdu, Sichuan, China; ^2^ Environment-friendly Crop Germplasm Innovation and Genetic Improvement Key Laboratory of Sichuan Province, Sichuan Academy of Agricultural Sciences, Chengdu, China

**Keywords:** vesicle trafficking, endocytosis, exocytosis, plant development, stress tolerance

## Abstract

The vesicle trafficking apparatus is a fundamental machinery to maintain the homeostasis of membrane-enclosed organelles in eukaryotic cells. Thus, it is broadly conserved in eukaryotes including plants. Intensive studies in the model organisms have produced a comprehensive picture of vesicle trafficking in yeast and human. However, with respect to the vesicle trafficking of plants including rice, our understanding of the components and their coordinated regulation is very limited. At present, several vesicle trafficking apparatus components and cargo proteins have been identified and characterized in rice, but there still remain large unknowns concerning the organization and function of the rice vesicle trafficking system. In this review, we outline the main vesicle trafficking pathways of rice based on knowledge obtained in model organisms, and summarize current advances of rice vesicle trafficking. We also propose to develop methodologies applicable to rice and even other crops for further exploring the mysteries of vesicle trafficking in plants.

## Introduction

The vesicle trafficking system is critical for the growth and development of eukaryotes since it is a fundamental machinery to maintain the homeostasis of membrane-enclosed organelles. Generally, this system is composed of the endoplasmic reticulum (ER), Golgi apparatus, vacuole, plasma membrane (PM), and intermediate organelles or vesicles (Aniento et al., 2021). Different types of vesicles, which initialize from distinct membranes, mediate cargo transport to various destinations for proper functions. There usually exist five kinds of vesicle-mediated cargo trafficking pathways in eukaryotes. The first type [coat protein I (COPI)-coated vesicles] and the second type (COPII-coated vesicles) are both involved in ER-Golgi trafficking; the third type [clathrin-coated vesicles (CCVs)] mediates endocytosis and post-Golgi trafficking; the fourth type [pre-vacuolar compartments (PVCs)/multivesicular bodies (MVBs)] modulates endocytosis, exocytosis, recycle, and degradation; and the fifth type [exocyst positive organelles (EXPO)] regulates exocytosis (Jürgens, 2004; Inada and Ueda, 2014; Wang W. -M. et al., 2016; Aniento et al., 2021). These vesicle-mediated trafficking pathways are usually divided into sequential steps including cargo selection, vesicle assembly/biogenesis, transportation, tethering and fusion, and cargo release (More et al., 2020).

Plants are sessile species that cannot move spontaneously. This fact is concomitant with many differences in the biological characteristics between plants and other species, with the vesicular system and the vesicle trafficking pathway being one of the main differences. For instance, plant cells lack the intermediate compartment, called ERGIC (ER-Golgi intermediate compartment), between the ER and the Golgi apparatus that are commonly found in mammals; plant seeds contain specialized vesicle trafficking pathways to mediate storage proteins trafficking into the protein storage vacuoles (PSVs) (Robinson et al., 2005; Gomez-Navarro and Miller, 2016). However, our knowledge regarding vesicle trafficking in plants including rice is rather limited compared to that obtained in mammals, which has heavily hampered the effort in manipulating vesicle trafficking for improving agronomic traits and crop production.

**Figure 1:**
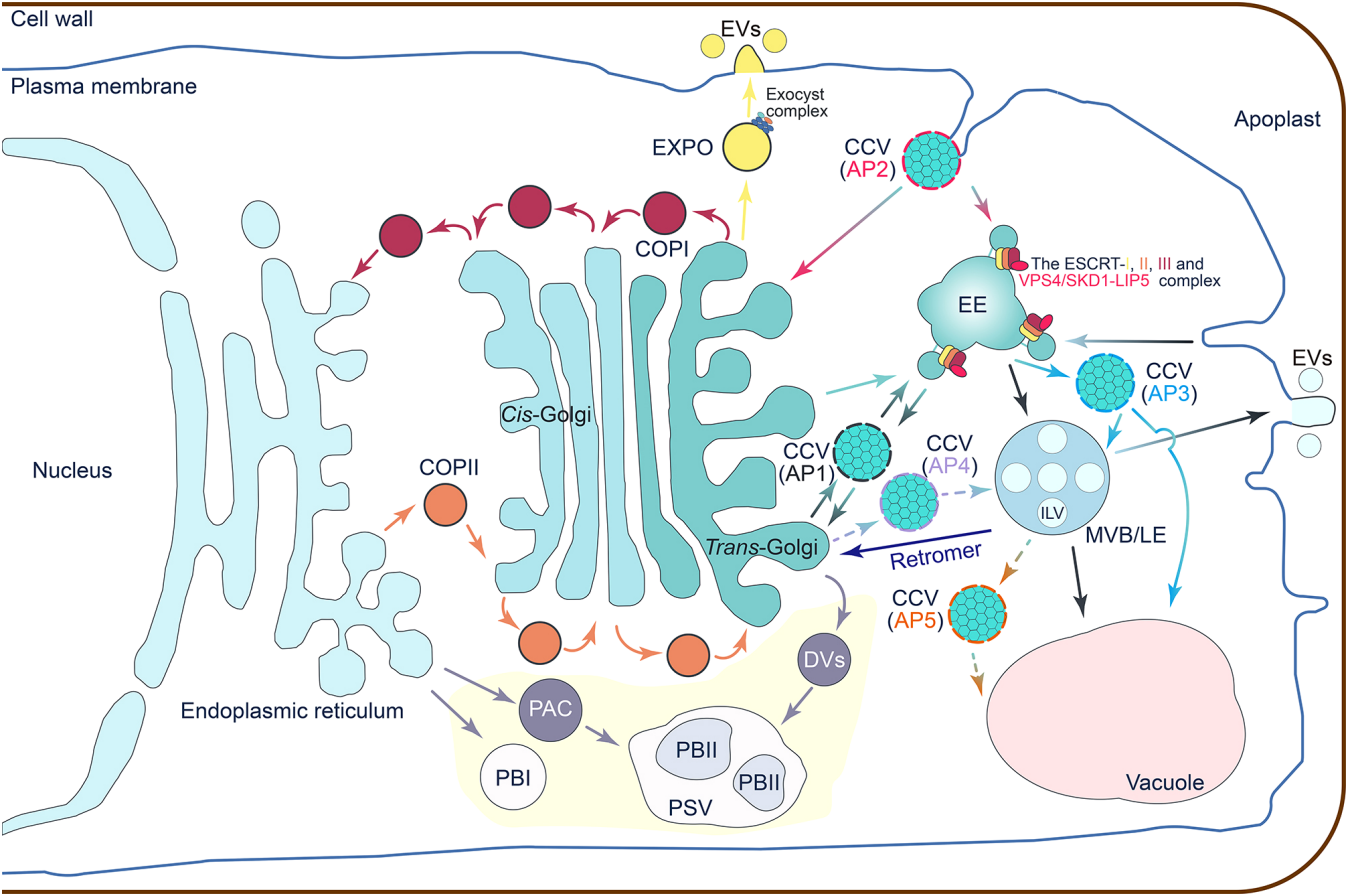
A schematic illustration that summarizes the vesicle trafficking network. The coat protein I (COPI)-coated vesicles are assembled on the Golgi apparatus to mediate retrograde trafficking from *cis*-Golgi back to the endoplasmic reticulum (ER), or intra-Golgi transport between different compartments of the Golgi apparatus. The COPII-coated vesicles are responsible for cargo recruitment and anterograde transport from ER to the Golgi apparatus. In the clathrin-coated vesicles (CCVs)-mediated trafficking routes, the adaptor protein complex 1 (AP1) positive CCVs are likely involved in trafficking between early endosomes (EEs) and the *cis*-Golgi network (TGN); the AP2 CCVs have a function in clathrin-mediated endocytosis of cargoes from the plasma membrane (PM); the AP3 CCVs mediate the transportation of cargo proteins from EEs to the late endosomes (LEs), lysosomes, and related organelles; the AP4 CCVs traffic cargoes from the TGN to the LEs; and the AP5 CCVs may be involved in protein trafficking from LEs to other membranous organelles such as vacuoles. The multivesicular body (MVB)-mediated vesicular trafficking is a hub for cargo endocytosis, exocytosis, recycling, and degradation. Cargo recognition and MVB maturation are mediated by the endosomal sorting complex required for transport (ESCRT) complexes consisting of ESCRT-I, -II, and -III sub-complexes, and the vacuolar protein sorting 4 (VPS4)/suppressor of K^+^ transport growth defect 1 (SKD1) complex. As an additional regulatory complex, the retromer complex controls the delivery of cargo from MVBs back to the TGN. The exocyst positive organelle (EXPO) vesicles mediate cargo exocytosis. The octameric protein complex and exocyst complex are involved in vesicle tethering and fusion. Storage proteins trafficking to protein storage vacuoles (PSVs) specifically occur in the endosperm and embryonic tissues (highlighted in a light-yellow background). Storage proteins synthesized in the ER may load and bud off directly from the ER to form the spherical protein body Is (PBIs). The storage proteins can also be transported from ER to PSVs by the precursor-accumulating vesicle (PAC) bypass of the Golgi apparatus to develop into the irregularly shaped PBIIs in PSVs. More commonly, storage proteins are passed through the Golgi apparatus and transported by the dense vesicles (DVs) to the PSVs and then develop into PBIIs. EVs, exosomes.

**Figure 2:**
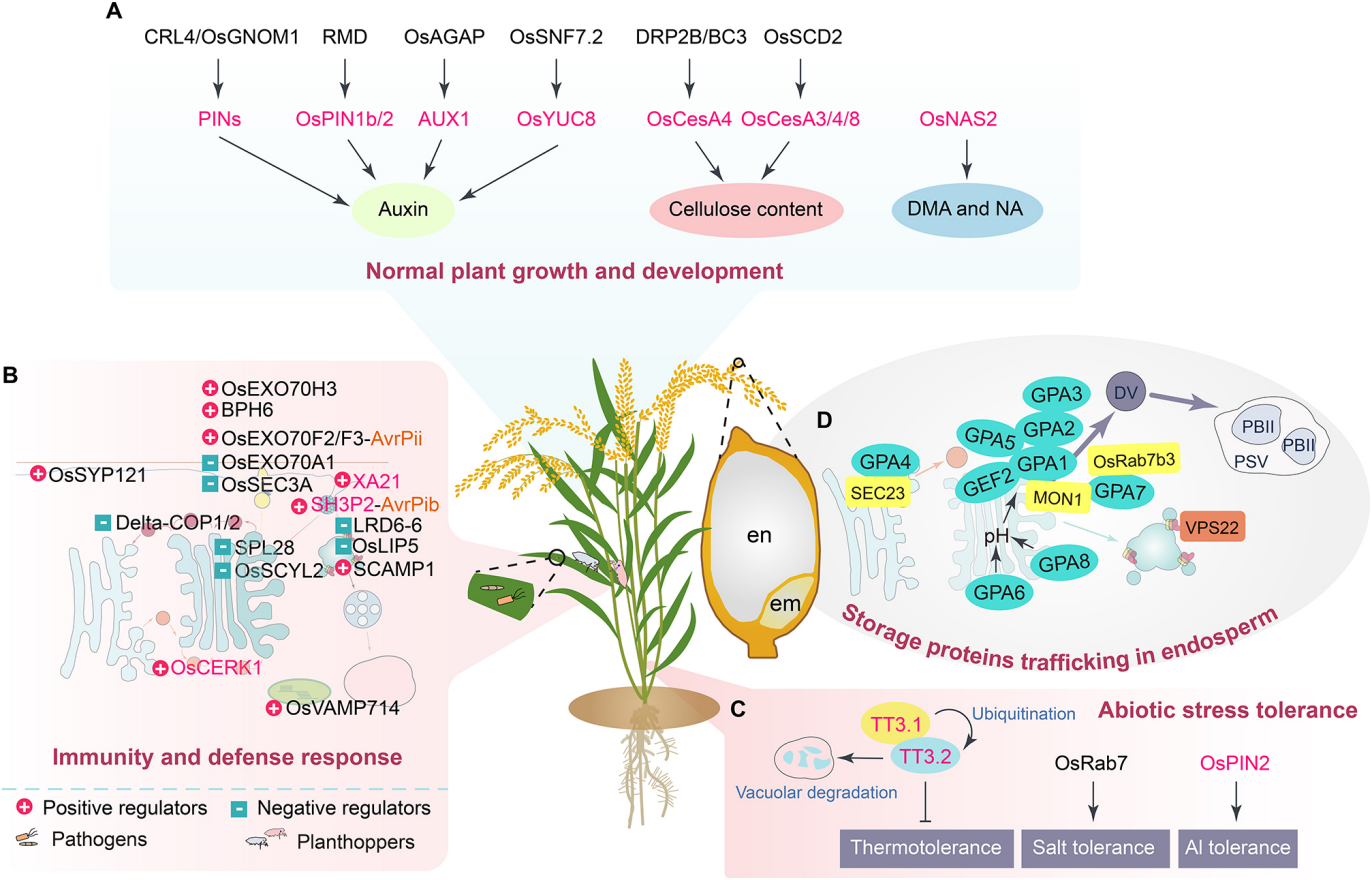
A schematic illustration summarizes the roles of currently known components and cargoes of the vesicle trafficking system in rice. **(A)** The proteins and pathways involved in regulating growth and development of rice. The known proteins associated with vesicle trafficking mainly function in auxin, cellulose, deoxymugineic acid (DMA), and nicotianamine (NA) biosynthesis pathways. **(B)** The elements and routes crucial for establishing rice immunity and defense responses. The regulatory roles of the proteins are indicated in the figure. **(C)** The proteins and manners associated with the regulation of rice abiotic stress tolerance. **(D)** The regulators and ways of controlling the trafficking of rice endosperm storage proteins. Glutelin precursor accumulation 1 (GPA1) is known as OsRab5a and Glutelin precursor mutant 4 (GLUP4), GPA2 is OsVPS9a and GLUP6, GPA4 is also termed as Golgi transport 1B (GOT1B) and GLUP2, GPA6 is named Rice Na^+^/H^+^ antiporter 5 (OsNHX5), GPA7 is Calcium caffeine zinc sensitivity 1 (CCZ1), and GPA8 is also named Rice vacuolar H^+^-ATPase (OsVHA). en, endosperm; em, embryo. The fonts in black indicate the key components of the vesicle trafficking apparatus, while the fonts in magenta represent cargo proteins.

Rice (*Oryza sativa* L.) is a staple crop feeding more than half of the world’s population. Thanks to the high quality of rice reference genome sequences, tremendous progresses have been achieved in understanding the molecular mechanisms and regulatory networks of various rice traits (Chen et al., 2022). Using sequence homology search, the typical vesicular trafficking components have been identified from rice genome and characterized for their functions, indicating that the rice plant is also equipped with the conserved vesicle trafficking pathways (Winter and Hauser, 2006; Zhao Z. et al., 2022). However, the crosstalk of vesicle trafficking pathways and the coordinate regulation of core elements are still largely unknown in rice. This review outlines the main vesicle trafficking pathways of rice based on achievements from model organisms (**Figure 1**) and summarizes current understanding of rice vesicle trafficking concerning growth, stress tolerance, and storage proteins trafficking in endosperm cells (**Figure 2**; **Table S1**).

## An overview of the vesicle trafficking network

### COPI/II-mediated trafficking: ER-Golgi trafficking

The COPI- and COPII-coated vesicles are involved in the trafficking between the ER and the Golgi apparatus. In general, COPI vesicle mediates retrograde trafficking from *cis*-Golgi back to the ER, or intra-Golgi transport between different compartments of the Golgi apparatus (Jackson, 2014; Gomez-Navarro and Miller, 2016). The COPII vesicle is responsible for cargo recruitment and anterograde transport from the ER to the Golgi apparatus (Gomez-Navarro and Miller, 2016).

COPI is a heptameric coat complex (also called coatomer for coat protomer) conceptually considered as B subcomplex and F subcomplex (Gomez-Navarro and Miller, 2016). The B subcomplex consisted of α, β′, and ϵ subunits and likely works as a “cage”, while the F subcomplex comprises coatomer subunits β, γ, δ, and ζ to function as an “adaptor” (Fiedler et al., 1996; Beck et al., 2009; Jackson, 2014; Aniento et al., 2021). For binding to different types of membrane proteins, both the B and the F subcomplexes are required and the subunits of these two subcomplexes are often coordinately cooperated, suggesting the complexity of the coatomer machinery (Beck et al., 2009; Jackson, 2014). The COPI vesicles is initiated by ADP-ribosylation factor 1 (ARF1), a small GTPase of the Ras superfamily. The cytosolic ARF1 in its GDP-bound state is recruited to the Golgi membranes through interacting with the p24 family proteins (Franco et al., 1996; Antonny et al., 1997; Liu Y. et al., 2009; Aniento et al., 2021). The guanine nucleotide exchange factor (GEF) of ARF1 (ARF1GEF) then induces the ARF1 to discharge its bound GDP and upload GTP, allowing the GTP-bound ARF1 to undergo conformational change for exposing its myristoylated N-terminal amphipathic helix, which helps ARF1 to insert into the membrane (Serafini et al., 1991; Franco et al., 1996; Beck et al., 2009). Subsequently, the coatomer subunits interact with ARF1-GTP and cargoes to form the COPI vesicles (Beck et al., 2009; Aniento et al., 2021). When the COPI vesicle reaches the destination membrane, the coat must be shed to allow for vesicle to fuse with the target membrane. This process is achieved by GTP hydrolysis in ARF1, which is catalyzed by the ARF1 GTPase-activating protein (ARF1GAP) (Tanigawa et al., 1993; Béthune and Wieland, 2018). Thus, the GTP exchange and hydrolysis, which are respectively promoted by ARF GEF/GAP, are important and responsible for both coatomer recruiting/uncoating and cargo packaging/releasing (Beck et al., 2009; Aniento et al., 2021).

COPI is involved in sorting many important retrograde and recycling cargoes. These include the K(H)DEL receptors recognized by ER Retention Defective 2 (ERD2) (Lewis and Pelham, 1990; Gaynor et al., 1994; Robinson and Aniento, 2020), the soluble N-ethylmaleimide-sensitive-factor attachment protein receptors (SNAREs) required in anterograde and retrograde pathways (Lewis and Pelham, 1996; Andag and Schmitt, 2003), the p24 family proteins with diphenylalanine motifs (FFXX, X represents any amino acid residue) (Fiedler et al., 1996; Sohn et al., 1996; Goldberg, 2000), the type I transmembrane proteins harboring C-terminal di-lysine based motifs [K(X)KXX] (Cosson and Letourneur, 1994; Letourneur et al., 1994), the type II transmembrane proteins lacking known coatomer binding motifs but interacting with coatomer and a coat adaptor VPS75p (Vacuolar protein sorting-associated protein 75) (Schmitz et al., 2008; Tu et al., 2008), and the ER proteins bearing the Arginine (Arg)-based motifs [(Φ/Ψ/R)RXR, Φ/Ψ is an aromatic or bulky hydrophobic residue] binding with coatomer subunits (Michelsen et al., 2005).

The COPII coat consists of five proteins, Secretion-associated and Ras-related 1 (SAR1), Secretory 23 (SEC23), SEC24, SEC13, and SEC31 (Sato and Nakano, 2007; Gomez-Navarro and Miller, 2016; Aniento et al., 2021). COPII coat assembly is initiated when the Ras superfamily small GTPase SAR1 is recruited and activated by the ER-localized transmembrane protein SEC12, which is a SAR1-specific GEF (Gomez-Navarro and Miller, 2016; Béthune and Wieland, 2018; Aniento et al., 2021). In the GTP-bound state, SAR1 exposes an N-terminal amphipathic α-helix to embed into the lipid bilayer of ER, leading to the membrane deformation and sequential recruitment of the COPII coat heterocomplexes (SEC23/SEC24 and SEC13/SEC31) (Aniento et al., 2021). The SEC23/SEC24 complex acts as a COPII adaptor complex. The hetero-tetramer SEC13/SEC31 is recruited to promote the formation of the COPII coated vesicles and the creation of a cage-like lattice with a cuboctahedral shape via the interaction between SAR1–SEC23 and SEC31 (Sato and Nakano, 2007; Aniento et al., 2021). SEC23 is the first protein recruited by activated SAR1 and SEC24, and it is required for cargo selection by directly interacting with the cargo proteins through the ER export signals such as the di-acidic motif [(D/E)X(D/E)], di-hydrophobic motifs (FF, YY, LL, or FY), and YXXXNPF and LXXME motifs (Sato and Nakano, 2007; Aniento et al., 2021). Soluble secretory proteins can also enter into COPII by the recognition of receptor proteins or as part of fluids and membranes (Sato and Nakano, 2007; Saito et al., 2017). At the *cis*-Golgi membrane, the GAP SEC23 will also execute GTPase activity to aid the GTP hydrolysis of SAR1 for achieving COPII uncoating (Sato and Nakano, 2007).

### CCV-mediated trafficking: endocytosis and post-Golgi trafficking

The CCV is another important coated vesicle responsible for endocytosis and many post-Golgi trafficking processes. CCVs are formed at the plasma membrane (PM), *trans*-Golgi network (TGN), and endosomes (Robinson, 2015). A typical CCV is assembled into a lattice of hexagons and pentagons, in which the outermost shell is a cage of interlocking clathrin triskelions that are built by three heavy and three light chains (Robinson and Pimpl, 2014; Robinson, 2015). The clathrin is recruited onto the membranes by the adaptor protein (AP) complex that confers to cargo selection (Robinson and Pimpl, 2014). There are five AP complexes (AP1–AP5) characterized to define five types of CCVs functioning in diverse trafficking pathways (Robinson and Pimpl, 2014; Robinson, 2015).

Among the five AP complexes, the AP2 complex-mediated CCV formation and transportation is the most thoroughly characterized one in clathrin-mediated endocytosis. It is a hetero-tetramer consisting of two large subunits/adaptins of approximately 100 kDa (α and β2), a medium-sized subunit of approximately 50 kDa (μ2), and a small subunit of approximately 20 kDa (δ2) (Boehm and Bonifacino, 2001; Robinson, 2015). The μ2 subunit is crucial for cargo selection because it can recognize both tyrosine-based (YXXF) and di-leucine based [(DE)XXXL(LI)] motifs in the cytosolic domains of transmembrane receptors (Kirchhausen, 2000; Robinson and Pimpl, 2014).

The biogenesis of the endocytic CCV can be briefly summarized as follows: (i) the AP2 complex is recruited to the PM by the α subunit via binding to the phosphatidylinositol 4,5-bisphosphate [PI(4,5)P2] on the PM; (ii) the μ2 subunit interacts with cargo or cargo receptors to promote the vesicle maturation efficiency; (iii) the clathrin triskelions and the remaining AP2 subunits are recruited to the PM; and (iv) the coated patch continues to grow and the dynamin is recruited to facilitate scission of CCVs from the PM (Robinson and Pimpl, 2014; Robinson, 2015). After scission, the uncoating machinery is recruited immediately for reasons that remain unknown (Robinson, 2015).

The remaining four AP complexes are also assembled similar to that of AP2 (Hirst et al., 2011; Robinson and Pimpl, 2014). Each of the AP complexes has a distinct localization and functions in different post-Golgi trafficking pathways. AP1 is found on early endosomes (EEs) and/or the TGN, indicating its involvement in trafficking among these two organelles (Hirst et al., 2011; Robinson and Pimpl, 2014; Robinson, 2015). AP3 transports cargoes from EEs to the late endosomes (LEs), lysosomes, and related organelles (Hirst et al., 2011; Robinson and Pimpl, 2014; Robinson, 2015). AP4 mobilizes cargo proteins from the TGN to LEs, while AP5 may be associated with protein trafficking from LEs to other membranous organelles such as vacuole (Hirst et al., 2011; Robinson and Pimpl, 2014; Robinson, 2015). Intriguingly, AP3 and AP4 are reported to work without clathrin in some cases (Boehm and Bonifacino, 2001; Robinson and Bonifacino, 2001).

### The MVB-mediated trafficking: endocytosis, exocytosis, recycle, and degradation

The MVBs, also known as PVCs and LEs, are derived and maturated from EEs (Cui et al., 2016). They are single membrane-bound organelles that function as core converging station of the secretory and endocytic pathways for cargo trafficking in eukaryotic cells (Wollert and Hurley, 2010; Cui et al., 2016). Both the newly synthesized proteins from TGN and the intrinsic PM-localized proteins can enter MVBs and are delivered into different destinations to execute proper functions (Wollert and Hurley, 2010; Cui et al., 2016). On one side, cargoes in MVBs can be sorted to the PM or released into the extracellular space once MVBs fuse with the PM. On the other side, cargoes in MVBs can be delivered into vacuole for degradation. Furthermore, cargoes can be recycled between PM and MVBs, or sent back to the TGN with the aid of the retromer complex (Wollert and Hurley, 2010; Cui et al., 2016; Li et al., 2018).

Cargo recognition and maturation of MVBs are mediated by the endosomal sorting complex required for transport (ESCRT) and Vacuolar protein sorting 4 (VPS4)/Suppressor of K+ transport growth defect 1 (SKD1)–LYST–interacting protein 5 (LIP5) complex (Henne et al., 2011). There are four conserved ESCRT (ESCRT-0, -I, -II, and -III) complexes in yeast and mammalian cells (Hurley, 2010; Henne et al., 2011; Cui et al., 2016). The ESCRT-0 complex recognizes the ubiquitination signal in the target proteins to initiate the sorting of cargoes. The ESCRT-I, -II, and -III complexes are then sequentially recruited to coordinately drive vesicle maturation (Wollert and Hurley, 2010; Henne et al., 2011). The final step in MVB maturation is the assembly of the VPS4/SKD1-LIP5 complex to ESCRT-III at the neck region of the limiting membrane. This facilitates neck scission, cargo packaging into the intralumenal vesicles (ILVs), and disassembly of the ESCRT-III complex (Wollert and Hurley, 2010; Henne et al., 2011).

The four ESCRT complexes constitute a precisely controlled process. The ESCRT-I complex functions to cluster the ubiquitinated cargo proteins and acts as a bridge for ESCRT-0 and ESCRT-II complexes (Henne et al., 2011; Cui et al., 2016). ESCRT-I works together with ESCRT-II at the limiting membrane to create and stabilize a vesicle neck. The ESCRT-II then recruits ESCRT-III to drive vesicle budding (Wollert and Hurley, 2010; Henne et al., 2011). While the ESCRT-0, -I, and -II complexes are stable protein complexes, the ESCRT-III transiently assembles on the endosome membrane (Wollert and Hurley, 2010; Henne et al., 2011). The ESCRT-III subunits can interact with deubiquitinating enzymes that catalyze the deubiquitylation of the cargo proteins (Isono et al., 2010; Cui et al., 2016). It is worth noting that the ESCRT-0 complex responsible for cargo selection has not been detected in plant. Instead, FYVE domain protein required for endosomal sorting 1 (FREE1) and Target of Myb 1 (TOM1) may execute the similar roles of the ESCRT-0 complex in plant, because these proteins can bind ubiquitin and interact with ESCRT subunits (Korbei et al., 2013; Gao et al., 2014; Cui et al., 2016).

### The EXPO-mediated exocytosis and possible roles in autophagy

The exocyst complex, termed EXPO, forms another exocytosis vesicle with unique double membrane structure distinct from MVBs (Wang J. et al., 2010; Wu and Guo, 2015). EXPO mainly mediates cargo transportation from TGN to the extracellular region by fusion with the PM (Wu and Guo, 2015). The exocyst is an octameric protein complex consisting of SEC3, SEC5, SEC6, SEC8, SEC10, SEC15, EXO70, and EXO84. Except for SEC3, the remaining seven subunits are associated with the vesicle membrane. SEC3 and EXO70 subunits can be associated with the PM through their binding ability with PI(4,5)P_2_ respectively endowed by polybasic sequences at the N-terminal of SEC3 and C-terminal domains of EXO70 (Wang J. et al., 2010; Ding et al., 2014; Wu and Guo, 2015). This interaction mediates the tethering of the EXPO to the PM, and the SNARE proteins then initiate the fusion of EXPO with the PM to release a single membrane vesicle termed exosome (EV) (Ding et al., 2014; Wu and Guo, 2015).

Exocyst assembly and function are modulated by different small GTPases from the Rab, Rho, and Ral families. In addition, subunits of the exocyst are targeted by a number of kinases for function (Wu and Guo, 2015). It is worth noting that besides their involvement in exocytosis, the exocyst complex can also function in many other biological processes such as cell migration, cytokinesis, and autophagy, which are either dependent or independent on their exocytosis-related roles (Wu and Guo, 2015; Žárský, 2022).

Autophagy is a highly conserved self-degradation mechanism for eukaryotes (Nakatogawa, 2020; Wang S. et al., 2022). The autophagy process can be divided into distinct stages including induction, cargo recognition, phagophore formation, phagophore expansion and closure, and autophagosome fusion with vacuole and breakdown of the inclusion (Nakatogawa, 2020; Su et al., 2020). More than 40 *autophagy-related genes* (*ATGs*) have been characterized in eukaryotes, and the ATG proteins can be divided into four functional groups as summarized by Su et al. (2020): (і) the ATG1/ATG13 kinase complex that initiates autophagosome formation; (ii) the autophagy-specific class III PI3-kinase complex; (iii) the ATG9 complex that promotes phagophore expansion; and (iv) the ATG8/ATG12 ubiquitin-like conjugation systems that act during phagophore expansion and maturation.

Interestingly, as mentioned above, specific exocyst subcomplexes have been found to engage in autophagy in both metazoans and plants (Bodemann et al., 2011; Kulich et al., 2013; Lin et al., 2021). For example, the Ras-like small G protein RalB and its effector EXO84 are required for nutrient starvation-induced autophagy in mammalian cells (Bodemann et al., 2011). Studying of autophagosome biogenesis in yeast also found that exocyst subunits except SEC15, EXO70, and EXO84 are involved in yeast autophagy (Singh et al., 2019). In plant, the exocyst subunit EXO70B1 is proved to be involved in autophagy because *Atexo70B1* exhibits starvation-dependent spontaneous leaf necrotic lesions like other autophagy mutants (Kulich et al., 2013). EXO70B1 interacts with the core exocyst subunit SEC5, which has been mechanistically implicated in autophagy regulation through interaction with ROP8, a member of the plant-specific Rho GTPases (Kulich et al., 2013; Lin et al., 2021). The *exo70B1 sec5a* double mutants present enhanced autophagy phenotype, providing further genetic evidence in supporting the participation of the exocyst subcomplex in autophagy (Kulich et al., 2013). Furthermore, present studies also suggest that EXO70Ds may act as possible selective autophagy receptors and there may also be secretory autophagy in plants (Acheampong et al., 2020; Žárský, 2022). In plant, it is observed that the EXO70 subunit is expanded due to the presence of many homologs (Ding et al., 2014; Fujisaki et al., 2015; Žárský et al., 2019; Pečenková et al., 2020), further suggesting that the EXPO and exocyst may have many more functions that remain to be studied.

### Plant vacuolar trafficking and protein storage vacuoles

Plant vacuole is the largest organelle in a plant cell and it plays multiple functions essential for plant growth and development (Zhang et al., 2014). The vacuole can respond to different growth conditions and cellular signaling to control cell growth by regulating the vacuole turgor pressure. It is also the storage organelle for proteins, lipids, sugars, and other metabolites (Zhang et al., 2014). There are different types of vacuoles in plants and different types of vacuoles may co-exist in certain cell types. The prevailing view is that the two types of vacuoles, lytic vacuoles (LVs) and protein storage vacuoles (PSVs), co-exist in cells of the developing embryo (Frigerio et al., 2008; Zouhar and Rojo, 2009). Only a single class of vacuoles is present, the central vacuoles/LVs in vegetative tissues (Zouhar and Rojo, 2009). In addition to LVs and PSVs, other types of vacuoles such as senescence-associated vacuoles (SAVs) have also been reported (Otegui et al., 2005), defining the diversity and complexity of plant vacuoles.

To serve their multiple functions, vacuoles must contain a precise complement of internal contents including proteins, lipids, and metabolites. Thus, transporting of cargoes into vacuole is well under organization and regulation. In general, there are three pathways, namely, the biosynthetic (a classical route), endocytic, and autophagic pathways that function in transportation of the cargoes, and even organelles into the vacuole (Zouhar and Rojo, 2009). In the biosynthetic and endocytic pathways, the post-Golgi trafficking apparatus including the ER, the Golgi apparatus, the TGN, the PVCs/MVBs, and the CCVs are utilized for sorting of cargoes from ER or PM to the vacuole, respectively (Brillada and Rojas-Pierce, 2017). The sorting receptors belong to the vacuole sorting receptor (VSR) or receptor homology region transmembrane domain RING-H2 (RMR) families and are required for recognition and selection of vacuolar cargoes (Brillada and Rojas-Pierce, 2017). The vacuolar cargo proteins usually contain one or more vacuolar sorting determinants (VSDs) that are recognized by sorting receptors (Zouhar and Rojo, 2009; Brillada and Rojas-Pierce, 2017). Two main types of VSDs have been identified, the sequence-specific VSDs (ssVSDs) and the C-terminal VSDs (ctVSDs). The ssVSDs show sequence conservation because they contain an Ile/Leu residue that is essential for vacuolar targeting (Zouhar and Rojo, 2009; Brillada and Rojas-Pierce, 2017). In contrast, the ctVSDs lack obvious sequence conservation, but they have their strict C-terminus localization of the protein and the over-representation of hydrophobic amino acids (Zouhar and Rojo, 2009; Brillada and Rojas-Pierce, 2017).

The cargo proteins loaded into the MVBs are then transported to and released into the vacuole. During the transportation process, many regulatory factors have been identified for their important roles (Brillada and Rojas-Pierce, 2017). These include the Rab GTPases from the Rab5 and Rab7 families (Uemura and Ueda, 2014), the AP3 and AP4 complexes (Xiang et al., 2013; Uemura and Ueda, 2014; Fuji et al., 2015), and the pH and regulatory lipids of the endomembrane organelles (Bassil et al., 2012; Schumacher, 2014). The last step of the vacuolar trafficking is the fusion of MVBs with the vacuole. The mechanisms of membrane fusion are highly conserved and mediated by the SNAREs and the HOPS (Homotypic fusion and vacuole protein sorting) tethering complexes (Xiang et al., 2013; Uemura and Ueda, 2014).

The autophagic pathway is a special pathway that can transport both cargoes and even organelles into the vacuole. Excess or harmful intracellular content or organelles can be encapsulated by the double-membrane autophagosome (summarized above) and transferred to vacuoles for degradation in plants (Nakatogawa, 2020; Wang S. et al., 2022). The autophagosomes are targeted to and fused with the vacuole under the precise regulation of the SNAREs and ESCRT complex (Su et al., 2020). Interestingly, the EXPO subunits such as EXO70B1, SEC5, and EXO70Ds are involved in autophagy-related transport to the vacuole (Kulich et al., 2013; Žárský, 2022), suggesting that the EXPO and autophagy may collaborate to regulate vacuolar transportation in certain conditions.

Protein storage vacuoles (PSVs) are typical storage compartments in endosperm of plant seeds. During seed maturation, large amounts of seed storage proteins are synthesized on the ER and delivered into vacuole until they are mobilized during germination (Shimada et al., 2018). There exist some differences in the trafficking machinery that deliver storage proteins to PSVs from that described above. For example, trafficking of several major PSV proteins from the Golgi apparatus into the PSVs is mediated by the dense vesicles (DVs) rather than MVBs and CCVs (Vitale and Hinz, 2005). DVs are small uniform vesicles approximately 150–200 nm in diameter that contain 7S and 11S globulins and may fuse directly to PSVs or develop into PSVs (Vitale and Hinz, 2005). The storage globulins belong to the 2S and 11S classes that can form electron-opaque aggregates in the ER and are sequestered into precursor-accumulating vesicles (PACs) and sent to PSVs by direct fusion with PSVs, bypassing the Golgi apparatus (Vitale and Hinz, 2005). The PACs are approximately 200 to 400 nm in diameter and contain an electron-dense core of storage proteins surrounded by an electron-translucent layer, and some vesicles also contained small vesicle-like structures (Hara-Nishimura et al., 1998). Interestingly, complex glycans are also found to associate with the peripheral region of PACs, suggesting that PACs may also be responsible for Golgi-derived glycoprotein storage and/or delivery (Hara-Nishimura et al., 1998).

In rice, three major types of storage proteins coexist in the endosperm, glutelins, prolamins, and α-globulins (Takemoto et al., 2002). Prolamins are synthesized in the ER and bud off from the ER as spherical protein body Is (PBIs) (Li et al., 1993). PBIs show a low electron density and a round shape with a diameter of 100–200 nm under electron microscopy, and the surface is surrounded by a layer of rough ER (Tanaka et al., 1980; Zhu et al., 2021). In contrast, protein body IIs (PBIIs) have a high electron density and they are irregular in shape, with a diameter of 300–400 nm (Tanaka et al., 1980; Zhu et al., 2021). PBIIs mainly accumulate glutelins and globulins (Tanaka et al., 1980). Glutelins are initially synthesized as 57-kDa proglutelins in the ER, then passed through the Golgi apparatus and transported by the DVs to the PSVs. In the PSVs, glutelins are cleaved into acidic and basic subunits by vacuolar processing enzymes, after which the PSVs develop into irregularly shaped PBIIs together with α-globulin (Robinson et al., 2005; Ren et al., 2014; Pan et al., 2021).

## The roles of vesicle trafficking in rice: important but poorly understood

### The vesicle trafficking system regulates growth and development of rice

The vesicle trafficking system has been reported to control normal growth and development of rice plant via coordinating the polar transport and biosynthesis of auxin, which is an important phytohormone modulating plant’s life cycle (Teale et al., 2006). The pin-formed (PIN) efflux carriers and the auxin-resistant (AUX) 1 influx carriers are two important auxin transport family members (Teale et al., 2006). The OsGNOM1 protein, a guanine nucleotide exchange factor for ADP-ribosylation factor, mediates auxin-dependent plant growth by coordinating the polar localization of auxin efflux carrier PIN1 (Kitomi et al., 2008; Liu S. et al., 2009). The mutant *crown rootless4* (*crl4*)*/osgnom1*, which resulted from mutation in OsGNOM1, is altered in the expression of genes *OsPIN2*, *OsPIN5b*, and *OsPIN9*, and displays impairment in auxin transport in shoots and roots, indicating that CRL4/OsGNOM1 affects the formation of adventitious roots through regulating of polar auxin transport (PAT) (Kitomi et al., 2008; Liu S. et al., 2009).

Auxin transport and signaling also depend on actin organization. *Rice Morphology Determinant* (*RMD*), a type II formin gene, is a crucial component in auxin-actin organization regulatory loop from the nucleus to cytoplasm required for rice growth and morphogenesis (Li et al., 2014). *RMD* is directly regulated by auxin response factor 23 (OsARF23) and OsARF24, and modulates endocytosis, exocytosis, and auxin-mediated OsPIN1b/2 recycling to the PM to affect auxin distribution and F-actin orientation (Li et al., 2014). Moreover, an ADP-ribosylation factor GTPase-activating protein (OsAGAP) also controls the vesicle transport route from PM to the Golgi apparatus, as well as the actin cytoskeletal organization (Du et al., 2011). Overexpression of *OsAGAP* impaired rice root development, likely due to the disruption of actin cytoskeleton and the concomitant increased endocytosis of AUX1 from PM (Du et al., 2011).

The sucrose non-fermenting protein (SNF) 7 is a component of the ESCRT-III complex that is involved in endosomal trafficking (Henne et al., 2011). A recent report shows that mutation of OsSNF7.2 resulted in adaxially rolled leaves due to the decreased number and size of the bulliform cells (Zhou et al., 2023). OsYUC8/Constitutively wilted 1 (COW1) is homologous to Arabidopsis YUCCA, which functions as an auxin biosynthesis enzyme known to control leaf rolling by regulating the size of bulliform cells (Woo et al., 2007; Zhou et al., 2023). In the regulation of rice leaf rolling, OsSNF7.2 interacts with OsYUC8 to aid its endosomal trafficking and vacuolar degradation (Zhou et al., 2023).

The plant cell wall is a basic cellular structure and primarily consists of polysaccharide polymers such as cellulose, hemicellulose, and pectin, as well as glycoproteins and lignin (Zhang et al., 2021). Cellulose synthase A (CesA) is involved in the biosynthesis of the crucial cell wall component cellulose (Zhang et al., 2021). In rice, dynamin-related protein (DRP) 2B/brittle culm 3 (BC3) is distributed on the PM, CCVs, and TGN to participate in the endocytic pathway and post-Golgi membrane trafficking pathway (Xiong et al., 2010). Mutation or overexpression of rice *DRP2B*/*BC3* altered the abundance of OsCesA4 in both the PM and the endomembrane system, and consequently changed the cellulose content and secondary wall structure (Xiong et al., 2010). The rice stomatal cytokinesis defective 2 (OsSCD2) is associated with clathrin and is responsible for normal function of endocytosis and post-Golgi trafficking (Wang F. et al., 2022). OsSCD2 plays important roles in maintaining rice normal growth, because its loss-of-function mutant affected the cellulose content likely due to the impaired trafficking of cellulose synthase OsCesA3, OsCesA4, and OsCesA8 (Wang F. et al., 2022).

Besides auxin and cellulose, the vesicle trafficking system is also potentially involved in deoxymugineic acid (DMA) and nicotianamine (NA) biosynthesis, and DMA secretion from rice roots (Nozoye et al., 2014). The rice NA synthase 2 (OsNAS2) is likely a cargo of the vesicle transport system because the amounts of NA and DMA are increased in plant overexpressing *OsNAS2-sGFP* (Nozoye et al., 2014). Moreover, Fe deficiency-inducible genes are upregulated when *OsNAS2-sGFP* is overexpressed, suggesting that OsNAS2 also regulates Fe homeostasis in rice although there remain many knowledge gaps (Nozoye et al., 2014).

### The vesicle trafficking system is crucial for rice stress tolerance

Environmental stresses, including biotic and abiotic stresses, cause severe yield losses in rice. Many rice improvement programs focus on the breeding of stress-tolerant varieties (Hernández-Soto et al., 2021). The conceptual understanding of how rice responds to biotic (e.g., pathogens and pests) and abiotic (e.g., heat and salinity) stresses is fundamental to the breeding programs. Therefore, the underlying molecular mechanisms have been extensively studied and many important progresses have been achieved (Hernández-Soto et al., 2021). Plants such as rice have evolved a two-layer surveillance system to detect pathogen invasion. In the first layer named pattern-triggered immunity (PTI), pattern recognition receptors (PRRs) on the plant PM sense microbe-derived immunogenic molecular patterns for activating defense responses (Zhao Y. et al., 2022). Adapted pathogens secrete virulence proteins called effectors into the host cells for successful invasion. In the second layer, plant intracellular receptors monitor the effectors to activate the more potent immune responses called effector-triggered immunity (ETI) (Zhao Y. et al., 2022).

In the arm race between pathogen and host, both sides have evolved effective strategies to utilize the vesicle trafficking system for their own purposes (Wang W. -M. et al., 2016). For example, the blast disease causal agent *Magnaporthe oryzae* can co-opt rice CCVs-mediated endocytosis to facilitate the delivery of fungal effectors into host cells for successful invasion (Oliveira-Garcia et al., 2023). Upon pathogen invasion, plants depend on vesicle trafficking system to timely deliver defense-associated molecules to right cellular compartments for establishing efficient immune responses. Furthermore, rice can monitor the integrity and accuracy of the vesicle trafficking machinery, because deficiency in the components of the vesicle trafficking system usually induces plant defense responses and cell death with elevated disease resistance. For example, knocking down the expression of the COPI component genes *Delta-COP1/2* leads to activated immune responses and cell death of rice plants (Ma et al., 2019). Loss of function of the TGN-derived CCV components, Spotted leaf mutant 28 (SPL28), Rice SCY1‐like 2 (OsSCYL2), and Rice clathrin heavy chain 1 (OsCHC1) similarly results in auto-immunity and cell death of the rice (Qiao et al., 2010; Yao et al., 2022). Mutation in the *Lesion resembling disease mutant 6-6* (*Lrd6-6*) encoding an AAA-type ATPase activates rice defense responses and promotes cell death due to impaired MVBs-mediated vesicular trafficking pathway (Zhu et al., 2016). OsLIP5 is a homolog of LIP5 from the VPS4/SKD1-LIP5 complex, and it may also function to facilitate MVB maturation (Henne et al., 2011). The *oslip5* mutant is severely stunted, develops necrotic lesions, and dies before flowering (Wang et al., 2023). This fact allows us to speculate that the *oslip5* mutant may display activated immunity despite the fact that its disease resistance remains unknown.

The exocyst complex also participates in modulating rice immunity. Loss of function of exocyst complex component OsSEC3A results in auto-immunity and cell death in rice (Ma et al., 2017), while mutation of OsEXO70A1 leads to ferruginous spotted necrotic leaves and alters growth and nutrient assimilation (Tu et al., 2015), indicating the negative regulatory roles of OsSEC3A and OsEXO70A1 on rice defense responses. In contrast, the components of the vesicle trafficking system may also function as positive regulators of rice defense responses and disease resistance, such as the SNARE protein Rice vesicle-associated membrane protein 714 (OsVAMP714), Rice syntaxin of plants 121 (OsSYP121), the EE localized SCAMP1 (Rice secretory carrier membrane protein 1), and the exocyst complex subunits OsEXO70F2/F3 (Chen F. et al., 2010; Fujisaki et al., 2015; Sugano et al., 2016; Cao et al., 2019).

Many immune-related proteins have been identified as cargo of the vesicle trafficking machinery in rice. XA21 is a receptor-like kinase protein that confers resistance to specific races of the bacterial blight disease pathogen *Xathomonas oryzae* pv. *oryzae* in rice (Song et al., 1995). During pathogen infection, the PM-localized XA21 can be internalized likely via the SCAMP1-positive EE pathway to initiate defense responses (Chen F. et al., 2010). The rice chitin elicitor receptor kinase 1 (OsCERK1) is a positive regulator of disease resistance (Shimizu et al., 2010), and its transportation from ER to the PM as a cargo of COPII trafficking pathway mediated by the small GTPase SAR1 is essential for rice immunity (Chen L. et al., 2010; Akamatsu et al., 2013). The endocytic CCVs provide spaces for recognition of AvrPib by Pib mediated by SH3 domain-containing protein 2 (SH3P2) to activate Pib-dependent ETI (Xie et al., 2022; Zhu and Chen, 2023). Moreover, the EXPO components OsEXO70F2/F3 can mediate the recognition of the AvrPii by Pii (Fujisaki et al., 2015; Xie et al., 2022). Collectively, the vesicular trafficking pathway is important for both PTI and ETI.

Except for determining a robust immunity against microbial pathogens, the vesicle trafficking apparatus, especially the EXPO pathway, is also required for rice to fight against pests. The brown planthopper (BPH) and the white-backed planthopper (WBPH) are the most destructive pests of rice (Guo et al., 2018). The BPH resistance gene *Bph6* encodes a protein that is localized to the exocyst. BPH6 interacts with OsEXO70E1 to promote exocytosis, such that it can maintain and reinforce plant cell wall for conferring broad resistance to planthoppers in rice (Guo et al., 2018). Another EXO family protein, OsEXO70H3, can also interact with BPH6 to mediate excretion of an *S*-adenosylmethionine synthetase-like protein (SAMSL) into apoplast to regulate lignin deposition in the cell wall, thus contributing to rice resistance to planthoppers (Wu et al., 2022).

The vesicle trafficking system has also been demonstrated to control rice abiotic stress tolerance. In the *thermo-tolerance 3* (*TT3*) gene locus, two genes *TT3.1* and *TT3.2* interact together to enhance rice thermotolerance and reduce yield losses caused by heat stress (Zhang et al., 2022). TT3.1 is a PM-localized RING-type E3 ligase, while TT3.2 is a chloroplast precursor protein that is imported from the cytosol to the chloroplasts. Upon heat stress, TT3.1 translocates to the endosomes where it ubiquitinates TT3.2 to promote TT3.2 vacuolar degradation (Zhang et al., 2022). Therefore, TT3.1 is considered as a potential thermosensor and lesser TT3.2 proteins imported into chloroplasts are beneficial for protecting thylakoids from heat stress (Zhang et al., 2022). The vesicle trafficking system has also been implicated to regulate salt and aluminum (Al) tolerance in rice, despite the fact that the molecular mechanism remains unknown. The overexpression of the small GTPase gene *OsRab7* increases the number of vesicles in rice root tip and enhances rice salt stress tolerance (Peng et al., 2014). Overexpressing *OsPIN2* enhances rice Al tolerance through elevating endocytic trafficking of Al from the cell wall to help internalize and sequestrate Al into cell sap in rice root tips (Wu et al., 2015).

### The vesicle trafficking system transports storage proteins in rice endosperm cells

Rice seeds accumulate large amounts of storage proteins including glutelins (approximately 80% of total seed storage proteins), prolamins, and α-globulin, which are important for seed germination and seedling growth (Zhu et al., 2021). There are two types of protein bodies, namely, PBI and PBII, which are responsible for protein storage in rice endosperm cells as described above. The components involved in regulating the delivery of storage proteins have been identified through analysis of *glutelin precursor accumulation* (*gpa*) mutants with abnormal endosperm in rice.

The GPA1 or GLUP4 (Glutelin precursor mutant 4) is a small GTPase OsRab5a associated with vesicle trafficking (Wang Y. et al., 2010). GPA1 functions in organizing the endomembrane system and plays an essential role in DV-mediated trafficking of storage proteins to PBII in PSVs (Wang Y. et al., 2010). GPA2, also named OsVPS9a or GLUP6, is a GEF for GPA1 to regulate storage protein trafficking in rice seeds (Liu et al., 2013). Another GEF of GPA1 is guanine nucleotide exchange factor 2 (GEF2), which functions cooperatively with GPA1 to control storage protein trafficking like that of GPA2 (Wen et al., 2015). GPA3 is a plant-specific kelch-repeat protein that directly interacts with GPA1 and GPA2 for storage proteins trafficking into PBII in PSVs (Ren et al., 2014). GPA5 is a plant-unique phox-homology domain-containing protein homologous to Arabidopsis endosomal Rab effector with PX-domain (Ren et al., 2020). As a membrane-associated protein specifically localized to DVs, GPA5 interacts and coordinates with GPA1, GPA2, and the Class C core vacuole/endosome tethering complex (CORVET)- and VAMP727-containing SNARE complexes to regulate DV-mediated post-Golgi trafficking of storage proteins to PBII in PSVs (Ren et al., 2020). GPA7, also named CCZ1, is a DUF1712 domain-containing protein homologous to Arabidopsis calcium caffeine zinc sensitivity1a (CCZ1a) and CCZ1b (Pan et al., 2021). GAP7 interacts with MON1 (Monensin sensitivity 1) to function as the Rab5 (e.g., GPA1) and Rab7 (e.g., OsRab7b3) GEF. Loss of function of GAP7 promotes the endosomal localization of GPA1 GEF GPA2. Thus, the GAP7-MON1 complex regulates DV-mediated trafficking of rice storage protein via the Rab5- and Rab7-dependent pathways (Pan et al., 2021). Collectively, GPA1/2/3/5/7, GEF2, MON1, and OsRab7b3 forms a complex to regulate DV-mediated rice storage proteins to PSVs in endosperm.

GPA6 and GPA8 are the other two GPA proteins functioning in storage proteins trafficking. GPA6 is a Na^+^/H^+^ antiporter called OsNHX5, while GPA8 is the subunit E isoform 1 of vacuolar H^+^-ATPase named Rice vacuolar H^+^-ATPase (OsVHA) (Zhu et al., 2019; Zhu et al., 2021). These two proteins regulate the endomembrane luminal pH to maintain Golgi morphology and the TGN localization of GPA1 and GPA3, thus conferring to storage protein trafficking in rice endosperm (Zhu et al., 2019; Zhu et al., 2021).

Two other storage protein trafficking pathways have been identified by characterizing the proteins GPA4 and VPS22. GPA4 is an evolutionarily conserved membrane protein also named GLUP2 and Golgi transport 1B (GOT1B). In rice, GPA4 interacts with SEC23 and resides in a complex containing SAR1b to mediate COPII vesicle formation at Golgi-associated ER exit sites (ERESs) (Fukuda et al., 2016; Wang Y. et al., 2016). GPA4 is required for localization of prolamine and α-globulin RNAs to the protein body ER and for efficient export of proglutelin and α-globulin proteins from the ER to the Golgi apparatus (Fukuda et al., 2016; Wang Y. et al., 2016). VPS22 is a component of the ESCRT-II complex, and its loss-of-function mutation leads to seedling death, and severe reduction in plant shoot and root growth (Zhang et al., 2013). The seeds of *vps22* mutant display chalky endosperm likely for impaired grain filling (Zhang et al., 2013), suggesting that the ESCRT-MVBs pathway is also involved in storage protein trafficking in rice endosperm cells.

## Conclusion

Despite the importance of vesicle trafficking for plants, the research progress of plant vesicle trafficking is rather limited compared to that of yeast and human. Our knowledge regarding plant vesicle trafficking is mainly derived from the model plant Arabidopsis, which is genetically and physiologically different from crop plants. In rice, the vesicle trafficking pathways are required for growth, defense responses, abiotic stress tolerance, and endosperm storage protein trafficking (**Figure 2**; **Table S1**). In recent years, some proteins associated with vesicle trafficking have been characterized in rice, offering many insights into the roles of vesicle trafficking in crops. Proteins such as OsGNOM1, OsSNF7.2, OsSEC3A, and LRD6-6 are components of vesicle trafficking apparatus, while OsYUC8 and OsCERK1 are cargoes of the vesicle trafficking system. However, rice vesicle trafficking is still poorly understood, not only for its molecular mechanism but also for our utilization of the knowledge to improve rice crop.

The reasons why it is so difficult to improve crops with current knowledge can be summarized as follows: (і) altering a vesicle trafficking pathway usually affects pleiotropic plant phenotype since a given vesicle trafficking pathway can transport numerous cargo proteins with diversity functions; (ii) it is difficult to utilize a specific cargo by simply manipulating the expression level of the coding gene, because transportation of a protein to the correct cellular site is not just governed by the amount of the protein. Nevertheless, at this moment, we believe that two strategies could be adopted to utilize vesicle trafficking to improve crops. One is to screen for excellent allelic mutations of the key genes involved in vesicle trafficking and then to utilize them using traditional or modern breeding methods. The other strategy is to use a tissue-specific or condition-inducible promoter to drive a gene or its valuable allele to promote it when needed to avoid adverse effects.

At the current stage, our knowledge of vesicle trafficking in rice is still not sufficient to support the breeding application, and there remains a long way to dissect the molecular mechanism of vesicle trafficking in rice. To systematically study and elucidate the vesicle trafficking apparatus in rice as well as other crops, the suitable technology and methodology are largely lacking at this moment. Thus, the first obstacle that must be solved is to develop and establish a methodology suitable for crops. This mainly includes methods concerning how to label, trace, and inhibit a specific type of vesicle and the corresponding trafficking pathway, and how to separate and purify different types of vesicles from plant cells or reconstitute the vesicle formation *in vitro*. Furthermore, it is common knowledge in crop plants that loss of function or even just reducing the expression levels of some genes in the vesicle trafficking system is lethal. Therefore, the methods for gene functional analysis require further improvement and new approaches are urgently needed. With the advancement in the methodology for studying the vesicle trafficking system in crops, we will be able to learn more about rice vesicle trafficking to allow for efficient genetic improvement of crop agronomic traits beneficial to human.

## Author contributions

XZ: Conceptualization, Funding acquisition, Writing – review & editing, Visualization, Writing – original draft. JY: Writing – original draft, Data curation, Investigation. HG: Data curation, Investigation, Writing – original draft. YW: Conceptualization, Funding acquisition, Project administration, Supervision, Writing – review & editing. BM: Conceptualization, Funding acquisition, Project administration, Supervision, Writing – review & editing.
