# Supplementary material for: Vesicle trafficking in rice: too little is known
Source: Front Plant Sci. 2023 Sep 18;14:1263966. doi: 10.3389/fpls.2023.1263966 (PMC10543891; doi:10.3389/fpls.2023.1263966)
Supplement: Supplementary file 1 [file Table_1.docx]

Supplementary Table 1. A summary of proteins that known to function as endomembrane trafficking system component or cargo in rice.

| **Protein Name** | **Protein type** | **Function description** | **References** |
| --- | --- | --- | --- |
| **Category 1**: Proteins responsible for rice normal growth and development. | | | |
| CRL4/OsGNOM1 | A GEF for ADP-ribosylation factor. | OsGNOM1 affects the formation of adventitious roots through regulating polar auxin transport. | (Kitomi et al., 2008;Liu et al., 2009) |
| RMD | A type II formin in rice. | RMD is required for endocytosis, exocytosis and auxin-mediated OsPIN1b/2 recycling to the plasma membrane to regulate auxin-mediated cell elongation, polar auxin transport, and auxin distribution gradients in root tips. Therefore, RMD is a crucial component that controls rice cell growth and morphogenesis. | (Li et al., 2014) |
| OsAGAP | A GAP for ADP-ribosylation factor. | Overexpressing *OsAGAP* stimulates vesicle transport and induces the accumulation of early endosomes and AUX1 to regulate rice root development. | (Du et al., 2011) |
| OsSNF7.2 | An ESCRT-III component. | OsSNF7.2 interacts with OsYUC8 and some other subunits of ESCRT-III to aid the vacuolar degradation of OsYUC8, conferring rice leaf development. | (Zhou et al., 2023) |
| DRP2B/BC3 | An authentic membrane-associated dynamin. | DRP2B/BC3 localizes at the PM, the CCVs and the TGN, conferring to the abundance OsCESA4 in the PM and in the endomembrane system, and thereby plays a critical role in secondary cell wall cellulose biosynthesis. | (Xiong et al., 2010) |
| OsSCD2 | A plant-specific protein that is homologous to *Arabidopsis* Stomatal Cytokinesis Defective 2. | OsSCD2 associates with clathrin to affect endocytosis and post-Golgi trafficking to play an important role in maintaining plant growth in rice. Loss function of OsSCD2 affects cellulose synthesis likely due to alteration in vesicular trafficking of OsCesA3/4/8. | (Wang et al., 2022) |
| OsNAS2 | A nicotianamine (NA) synthase. | OsNAS2 is likely localized to vesicular trafficking system. Overexpressing OsNAS2 upregulates Fe deficiency-inducible genes and synthesizes more amounts of NA and DMA. | (Nozoye et al., 2014) |
| **Category 2**: Proteins function in rice immunity and defense response. | | | |
| Delta-COP1/2 | COPI coatomer delta subunits. | Delta-COP1/2 interacts with SPL35 and negatively regulates rice immunity and cell death. | (Ma et al., 2019) |
| SPL28 | AP1 medium subunit μ1 of CCV. | SPL28 localizes to the Golgi apparatus and negatively regulates rice immunity and cell death. | (Qiao et al., 2010) |
| OsSCYL2 | The SCY1‐like family protein, a component of CCV. | OsSCYL2 interacts with OsCHC1 and SPL28 to negatively regulate rice immunity and cell death. | (Yao et al., 2022) |
| OsVAMP714 | A SNARE protein. | OsVAMP714 localizes to chloroplast and cellular periphery, and will translocate to the vacuolar membrane surrounding the invasive hyphae under *M. oryzae* invasion to positively regulates rice disease resistance. | (Sugano et al., 2016) |
| OsSYP121 | A SNARE protein. | OsSYP121 is a plasma membrane localized SNARE protein that interacts with OsSNAP32 and OsVAMP714, accumulating at the fungal penetration sites to contribute to disease resistance. | (Cao et al., 2019) |
| OsEXO70F2/F3 | The exocyst complex subunits. | OsEXO70F2/F3 interacts with the *M. oryzae* avirulent protein AvrPii to specifically regulate Pii mediated disease resistance. | (Fujisaki et al., 2015) |
| OsEXO70A1 | An exocyst complex subunit. | Loss-of-function mutation of OsEXO70A1 leads to irregular SCW deposition and ferruginous spotted necrotic leaves. | (Tu et al., 2015) |
| OsSEC3A | An exocyst complex subunit. | OsSEC3A interacts with the SNARE protein OsSNAP32 to negatively regulates rice immunity and cell death. | (Ma et al., 2017) |
| LRD6-6 | The AAA type ATPase homologous to VPS4/SKD1. | LRD6-6 associats with the ESCRT-III components OsSNF7 and OsVPS2 to facilitate the maturation of MVBs and negatively regulate rice immunity and cell death. | (Zhu et al., 2016) |
| OsCERK1 | Receptor kinase. | OsCERK1 is a chitin elicitor receptor kinase that positively regulates rice immunity and disease resistance, and it is a cargo of COPII. | (Chen et al., 2010b;Shimizu et al., 2010;Akamatsu et al., 2013) |
| SH3P2 | SH3 domain-containing protein. | SH3P2 interacts with Pib and AvrPib, displaying higher affinity to AvrPib. In detail, SH3P2 associates with Pib at CCVs to keep Pib in a static state and Pib-mediated resistance will be activated on recognition of AvrPib when infected by *M. oryzae* containing AvrPib. | (Xie et al., 2022;Zhu and Chen, 2023) |
| XA21 | Receptor kinase. | XA21 might be endocytosed via the SCAMP1-positive early endosomal trafficking to initiate resistance responses during pathogen infection. | (Lam et al., 2007;Chen et al., 2010a) |
| BPH6 | A protein has no similarity to known proteins that is localized to exocyst. | BPH6 interacts with OsEXO70E1 to promote exocytosis and participates in cell wall maintenance and reinforcement, conferring to broad resistance to planthoppers in rice. | (Guo et al., 2018) |
| OsEXO70H3 | An exocyst complex subunit. | OsEXO70H3 interacts with BPH6 and an *S*-adenosylmethionine synthetase-like protein (SAMSL) that may be involved in lignin deposition in the cell wall. OsEXO70H3 mediates cell excretion of SAMSL to apoplast to regulate lignin deposition in the cell wall, thus contributing to rice resistance to planthoppers. | (Wu et al., 2022) |
| **Category 3**: Proteins needed for rice abiotic stress tolerance. | | | |
| OsRab7 | A small GTPase involved in vesicle trafficking. | Overexpressing OsRab7 increases the number of vesicles in rice root tip and enhances plant salt stress tolerance. | (Peng et al., 2014) |
| OsPIN2 | A PIN-FORMED (PIN) protein family member. | Overexpressing OsPIN2 enhances rice aluminium (Al) tolerance through elevated endocytic vesicular trafficking and Al internalization and sequestration. | (Wu et al., 2015) |
| TT3 | Consisting of two genes, TT3.1 (a RING-type E3 ligase) and TT3.2 (a chloroplast precursor protein). | TT3.1 is a positive regulator to promote thermotolerance, whereas TT3.2 plays a negative role. Upon heat stress, plasma membrane–localized TT3.1 translocates to the endosomes, on which TT3.1 ubiquitinates TT3.2 for vacuolar degradation to enhance rice thermotolerance. | (Zhang et al., 2022) |
| **Category 4**: Proteins play important roles in storage proteins trafficking in rice endosperm cells. | | | |
| GPA1 | A small GTPase involved in vesicle trafficking. | GAP1 is also known as OsRab5a which functions in organizing of the endomembrane system and plays an essential role in trafficking of storage protein to PBII. | (Wang et al., 2010;Fukuda et al., 2011) |
| GPA2 | OsVPS9a, a GEF of OsRab5a. | VPS9a/ GLUP6 is an GEF of the small GTPase OsRab5a and works together with OsRab5a to regulate storage protein trafficking in rice seeds. | (Fukuda et al., 2013;Liu et al., 2013) |
| GEF2 | A GEF of OsRab5a. | Another GEF of the small GTPase OsRab5a in regulating of rice storage protein trafficking in seeds. | (Wen et al., 2015) |
| GPA3 | A plant-specific kelch-repeat protein. | GPA3 directly interacts with OsRab5a GEF VPS9a and forms a regulatory complex with OsRab5a via VPS9a to regulate rice storage protein trafficking in seeds. | (Ren et al., 2014) |
| GPA4 | An evolutionarily conserved membrane protein GOT1B (also known as GLUP2). | GPA4 interacts with SEC23 and presents in a complex with rice SAR1b to play important roles in mediating COPII vesicle formation at Golgi-associated ER exit sites (ERESs). It is required for localization of prolamine and α-globulin RNAs to the protein body ER and for efficient export of proglutelin and α-globulin proteins from the ER to the Golgi apparatus. | (Fukuda et al., 2016;Wang et al., 2016) |
| GPA5 | A plant-unique phox-homology domain-containing protein homologous to *Arabidopsis* endosomal Rab effector with px-domain. | GPA5 is a membrane-associated protein that is specifically localized to DVs. It interacts and coordinates with OsRab5a, VPS9a and CORVET-, VAMP727-containing SNARE complexes to regulate DV-mediated post-Golgi trafficking of rice storage proteins to PSVs in endosperm. | (Ren et al., 2020) |
| GPA6 | A Na^+^/H^+^ antiporter also named as OsNHX5. | OsNHX5 protein colocalizes to the Golgi, the TGN and the PVC to regulate the endomembrane luminal pH that is essential for seed storage protein trafficking in rice. | (Zhu et al., 2019) |
| GPA7 | A predicted DUF1712 domain containing protein homologous to Arabidopsis Calcium Caffeine Zinc Sensitivity1a (CCZ1a) and CCZ1b. | GAP7 interacts with MON1 and function together as the Rab5 effector and the Rab7 GEF. Loss of GAP7 function promoted the endosomal localization of OsRab5a GEF VPS9a. Thus, the GAP7-MON1 complex regulates post-Golgi trafficking of rice storage protein via the Rab5- and Rab7-dependent pathways. | (Pan et al., 2021) |
| GPA8 | OsVHA, subunit E isoform 1 of vacuolar H^+^ -ATPase. | GPA8 mainly localizes to TGN and the tonoplast. It regulates the luminal pH of the TGN and vacuole to maintain Golgi morphology and TGN localization of GPA1 and GPA3 thus conferring to storage protein trafficking in rice endosperm. | (Zhu et al., 2021) |
| VPS22 | A component of the ESCRT-II complex. | Loss of VPS22 function leads to seedling lethal, severe reduction in plant shoot and root growth. The seeds of *vps22* presents chalky endosperm likely for impaired grain filling. | (Zhang et al., 2013) |

**References**

Akamatsu, A., Wong, H.L., Fujiwara, M., Okuda, J., Nishide, K., Uno, K., Imai, K., Umemura, K., Kawasaki, T., Kawano, Y., and Shimamoto, K. (2013). An OsCEBiP/OsCERK1-OsRacGEF1-OsRac1 module is an essential early component of chitin-induced rice immunity. *Cell Host & Microbe* 13**,** 465-476. doi: 10.1016/j.chom.2013.03.007.

Cao, W.-L., Yu, Y., Li, M.-Y., Luo, J., Wang, R.-S., Tang, H.-J., Huang, J., Wang, J.-F., Zhang, H.-S., and Bao, Y.-M. (2019). OsSYP121 accumulates at fungal penetration sites and mediates host resistance to rice blast. *Plant Physiology* 179**,** 1330-1342. doi: 10.1104/pp.18.01013.

Chen, F., Gao, M.-J., Miao, Y.-S., Yuan, Y.-X., Wang, M.-Y., Li, Q., Mao, B.-Z., Jiang, L.-W., and He, Z.-H. (2010a). Plasma membrane localization and potential endocytosis of constitutively expressed XA21 proteins in transgenic rice. *Molecular Plant* 3**,** 917-926. doi: <https://doi.org/10.1093/mp/ssq038>.

Chen, L., Hamada, S., Fujiwara, M., Zhu, T., Thao, N.P., Wong, H.L., Krishna, P., Ueda, T., Kaku, H., Shibuya, N., Kawasaki, T., and Shimamoto, K. (2010b). The Hop/Sti1-Hsp90 chaperone complex facilitates the maturation and transport of a PAMP receptor in rice innate immunity. *Cell Host & Microbe* 7**,** 185-196. doi: 10.1016/j.chom.2010.02.008.

Du, C., Xu, Y., Wang, Y., and Chong, K. (2011). Adenosine diphosphate ribosylation factor-GTPase-activating protein stimulates the transport of AUX1 endosome, which relies on actin cytoskeletal organization in rice root development. *Journal of Integrative Plant Biology* 53**,** 698-709. doi: <https://doi.org/10.1111/j.1744-7909.2011.01059.x>.

Fujisaki, K., Abe, Y., Ito, A., Saitoh, H., Yoshida, K., Kanzaki, H., Kanzaki, E., Utsushi, H., Yamashita, T., Kamoun, S., and Terauchi, R. (2015). Rice Exo70 interacts with a fungal effector, AVR-Pii, and is required for AVR-Pii-triggered immunity. *The Plant Journal* 83**,** 875-887. doi: <https://doi.org/10.1111/tpj.12934>.

Fukuda, M., Kawagoe, Y., Murakami, T., Washida, H., Sugino, A., Nagamine, A., Okita, T.W., Ogawa, M., and Kumamaru, T. (2016). The dual roles of the Golgi transport 1 (GOT1B): RNA localization to the cortical endoplasmic reticulum and the export of proglutelin and α-Globulin from the cortical ER to the Golgi. *Plant and Cell Physiology* 57**,** 2380-2391. doi: 10.1093/pcp/pcw154.

Fukuda, M., Satoh-Cruz, M., Wen, L., Crofts, A.J., Sugino, A., Washida, H., Okita, T.W., Ogawa, M., Kawagoe, Y., Maeshima, M., and Kumamaru, T. (2011). The small GTPase Rab5a is essential for intracellular transport of proglutelin from the Golgi apparatus to the protein storage vacuole and endosomal membrane organization in developing rice endosperm. *Plant Physiology* 157**,** 632-644. doi: 10.1104/pp.111.180505.

Fukuda, M., Wen, L., Satoh-Cruz, M., Kawagoe, Y., Nagamura, Y., Okita, T.W., Washida, H., Sugino, A., Ishino, S., Ishino, Y., Ogawa, M., Sunada, M., Ueda, T., and Kumamaru, T. (2013). A guanine nucleotide exchange factor for Rab5 proteins is essential for intracellular transport of the proglutelin from the Golgi apparatus to the protein storage vacuole in rice endosperm. *Plant Physiology* 162**,** 663-674. doi: 10.1104/pp.113.217869.

Guo, J., Xu, C., Wu, D., Zhao, Y., Qiu, Y., Wang, X., Ouyang, Y., Cai, B., Liu, X., Jing, S., Shangguan, X., Wang, H., Ma, Y., Hu, L., Wu, Y., Shi, S., Wang, W., Zhu, L., Xu, X., Chen, R., Feng, Y., Du, B., and He, G. (2018). Bph6 encodes an exocyst-localized protein and confers broad resistance to planthoppers in rice. *Nature Genetics* 50**,** 297-306. doi: 10.1038/s41588-018-0039-6.

Kitomi, Y., Ogawa, A., Kitano, H., and Inukai, Y. (2008). CRL4 regulates crown root formation through auxin transport in rice. *Plant Root* 2**,** 19-28. doi: 10.3117/plantroot.2.19.

Lam, S.K., Siu, C.L., Hillmer, S., Jang, S., An, G., Robinson, D.G., and Jiang, L. (2007). Rice SCAMP1 defines clathrin-coated, trans-Golgi–located tubular-vesicular structures as an early endosome in tobacco BY-2 cells. *The Plant Cell* 19**,** 296-319. doi: 10.1105/tpc.106.045708.

Li, G., Liang, W., Zhang, X., Ren, H., Hu, J., Bennett, M.J., and Zhang, D. (2014). Rice actin-binding protein RMD is a key link in the auxin–actin regulatory loop that controls cell growth. *Proceedings of the National Academy of Sciences* 111**,** 10377-10382. doi: doi:10.1073/pnas.1401680111.

Liu, F., Ren, Y., Wang, Y., Peng, C., Zhou, K., Lv, J., Guo, X., Zhang, X., Zhong, M., Zhao, S., Jiang, L., Wang, H., Bao, Y., and Wan, J. (2013). OsVPS9A functions cooperatively with OsRAB5a to regulate post-Golgi dense vesicle-mediated storage protein trafficking to the protein storage vacuole in rice endosperm cells. *Molecular Plant* 6**,** 1918-1932. doi: <https://doi.org/10.1093/mp/sst081>.

Liu, S., Wang, J., Wang, L., Wang, X., Xue, Y., Wu, P., and Shou, H. (2009). Adventitious root formation in rice requires OsGNOM1 and is mediated by the OsPINs family. *Cell Research* 19**,** 1110-1119. doi: 10.1038/cr.2009.70.

Ma, J., Chen, J., Wang, M., Ren, Y., Wang, S., Lei, C., Cheng, Z., and Sodmergen (2017). Disruption of OsSEC3A increases the content of salicylic acid and induces plant defense responses in rice. *Journal of Experimental Botany* 69**,** 1051-1064. doi: 10.1093/jxb/erx458.

Ma, J., Wang, Y., Ma, X., Meng, L., Jing, R., Wang, F., Wang, S., Cheng, Z., Zhang, X., Jiang, L., Wang, J., Wang, J., Zhao, Z., Guo, X., Lin, Q., Wu, F., Zhu, S., Wu, C., Ren, Y., Lei, C., Zhai, H., and Wan, J. (2019). Disruption of gene SPL35, encoding a novel CUE domain-containing protein, leads to cell death and enhanced disease response in rice. *Plant Biotechnology Journal* 17**,** 1679-1693. doi: <https://doi.org/10.1111/pbi.13093>.

Nozoye, T., Tsunoda, K., Nagasaka, S., Bashir, K., Takahashi, M., Kobayashi, T., Nakanishi, H., and Nishizawa, N.K. (2014). Rice nicotianamine synthase localizes to particular vesicles for proper function. *Plant Signaling & Behavior* 9**,** e28660. doi: 10.4161/psb.28660.

Pan, T., Wang, Y., Jing, R., Wang, Y., Wei, Z., Zhang, B., Lei, C., Qi, Y., Wang, F., Bao, X., Yan, M., Zhang, Y., Zhang, P., Yu, M., Wan, G., Chen, Y., Yang, W., Zhu, J., Zhu, Y., Zhu, S., Cheng, Z., Zhang, X., Jiang, L., Ren, Y., and Wan, J. (2021). Post-Golgi trafficking of rice storage proteins requires the small GTPase Rab7 activation complex MON1–CCZ1. *Plant Physiology* 187**,** 2174-2191. doi: 10.1093/plphys/kiab175.

Peng, X., Ding, X., Chang, T., Wang, Z., Liu, R., Zeng, X., Cai, Y., and Zhu, Y. (2014). Overexpression of a vesicle trafficking gene, OsRab7, enhances salt tolerance in rice. *The Scientific World Journal* 2014**,** 483526. doi: 10.1155/2014/483526.

Qiao, Y., Jiang, W., Lee, J., Park, B., Choi, M.-S., Piao, R., Woo, M.-O., Roh, J.-H., Han, L., Paek, N.-C., Seo, H.S., and Koh, H.-J. (2010). SPL28 encodes a clathrin-associated adaptor protein complex 1, medium subunit μ1 (AP1M1) and is responsible for spotted leaf and early senescence in rice (*Oryza sativa*). *New Phytologist* 185**,** 258-274. doi: <https://doi.org/10.1111/j.1469-8137.2009.03047.x>.

Ren, Y., Wang, Y., Liu, F., Zhou, K., Ding, Y., Zhou, F., Wang, Y., Liu, K., Gan, L., Ma, W., Han, X., Zhang, X., Guo, X., Wu, F., Cheng, Z., Wang, J., Lei, C., Lin, Q., Jiang, L., Wu, C., Bao, Y., Wang, H., and Wan, J. (2014). GLUTELIN PRECURSOR ACCUMULATION3 encodes a regulator of post-Golgi vesicular traffic essential for vacuolar protein sorting in rice endosperm. *The Plant Cell* 26**,** 410-425. doi: 10.1105/tpc.113.121376.

Ren, Y., Wang, Y., Pan, T., Wang, Y., Wang, Y., Gan, L., Wei, Z., Wang, F., Wu, M., Jing, R., Wang, J., Wan, G., Bao, X., Zhang, B., Zhang, P., Zhang, Y., Ji, Y., Lei, C., Zhang, X., Cheng, Z., Lin, Q., Zhu, S., Zhao, Z., Wang, J., Wu, C., Qiu, L., Wang, H., and Wan, J. (2020). GPA5 encodes a Rab5a effector required for post-Golgi trafficking of rice storage proteins. *The Plant Cell* 32**,** 758-777. doi: 10.1105/tpc.19.00863.

Shimizu, T., Nakano, T., Takamizawa, D., Desaki, Y., Ishii-Minami, N., Nishizawa, Y., Minami, E., Okada, K., Yamane, H., Kaku, H., and Shibuya, N. (2010). Two LysM receptor molecules, CEBiP and OsCERK1, cooperatively regulate chitin elicitor signaling in rice. *The Plant Journal* 64**,** 204-214. doi: <https://doi.org/10.1111/j.1365-313X.2010.04324.x>.

Sugano, S., Hayashi, N., Kawagoe, Y., Mochizuki, S., Inoue, H., Mori, M., Nishizawa, Y., Jiang, C.-J., Matsui, M., and Takatsuji, H. (2016). Rice OsVAMP714, a membrane-trafficking protein localized to the chloroplast and vacuolar membrane, is involved in resistance to rice blast disease. *Plant Molecular Biology* 91**,** 81-95. doi: 10.1007/s11103-016-0444-0.

Tu, B., Hu, L., Chen, W., Li, T., Hu, B., Zheng, L., Lv, Z., You, S., Wang, Y., Ma, B., Chen, X., Qin, P., and Li, S. (2015). Disruption of OsEXO70A1 causes irregular vascular bundles and perturbs mineral nutrient assimilation in rice. *Scientific Reports* 5**,** 18609. doi: 10.1038/srep18609.

Wang, F., Cheng, Z., Wang, J., Zhang, F., Zhang, B., Luo, S., Lei, C., Pan, T., Wang, Y., Zhu, Y., Wang, M., Chen, W., Lin, Q., Zhu, S., Zhou, Y., Zhao, Z., Wang, J., Guo, X., Zhang, X., Jiang, L., Bao, Y., Ren, Y., and Wan, J. (2022). Rice STOMATAL CYTOKINESIS DEFECTIVE2 regulates cell expansion by affecting vesicular trafficking in rice. *Plant Physiology* 189**,** 567-584. doi: 10.1093/plphys/kiac073.

Wang, Y., Liu, F., Ren, Y., Wang, Y., Liu, X., Long, W., Wang, D., Zhu, J., Zhu, X., Jing, R., Wu, M., Hao, Y., Jiang, L., Wang, C., Wang, H., Bao, Y., and Wan, J. (2016). GOLGI TRANSPORT 1B regulates protein export from the endoplasmic reticulum in rice endosperm cells. *The Plant Cell* 28**,** 2850-2865. doi: 10.1105/tpc.16.00717.

Wang, Y., Ren, Y., Liu, X., Jiang, L., Chen, L., Han, X., Jin, M., Liu, S., Liu, F., Lv, J., Zhou, K., Su, N., Bao, Y., and Wan, J. (2010). OsRab5a regulates endomembrane organization and storage protein trafficking in rice endosperm cells. *The Plant Journal* 64**,** 812-824. doi: <https://doi.org/10.1111/j.1365-313X.2010.04370.x>.

Wen, L., Fukuda, M., Sunada, M., Ishino, S., Ishino, Y., Okita, T.W., Ogawa, M., Ueda, T., and Kumamaru, T. (2015). Guanine nucleotide exchange factor 2 for Rab5 proteins coordinated with GLUP6/GEF regulates the intracellular transport of the proglutelin from the Golgi apparatus to the protein storage vacuole in rice endosperm. *Journal of Experimental Botany* 66**,** 6137-6147. doi: 10.1093/jxb/erv325.

Wu, D., Guo, J., Zhang, Q., Shi, S., Guan, W., Zhou, C., Chen, R., Du, B., Zhu, L., and He, G. (2022). Necessity of rice resistance to planthoppers for OsEXO70H3 regulating SAMSL excretion and lignin deposition in cell walls. *New Phytologist* 234**,** 1031-1046. doi: <https://doi.org/10.1111/nph.18012>.

Wu, D., Shen, H., Yokawa, K., and Baluška, F. (2015). Overexpressing OsPIN2 enhances aluminium internalization by elevating vesicular trafficking in rice root apex. *Journal of Experimental Botany* 66**,** 6791-6801. doi: 10.1093/jxb/erv385.

Xie, Y., Wang, Y., Yu, X., Lin, Y., Zhu, Y., Chen, J., Xie, H., Zhang, Q., Wang, L., Wei, Y., Xiao, Y., Cai, Q., Zheng, Y., Wang, M., Xie, H., and Zhang, J. (2022). SH3P2, an SH3 domain-containing protein that interacts with both Pib and AvrPib, suppresses effector-triggered, Pib-mediated immunity in rice. *Molecular Plant* 15**,** 1931-1946. doi: <https://doi.org/10.1016/j.molp.2022.10.022>.

Xiong, G., Li, R., Qian, Q., Song, X., Liu, X., Yu, Y., Zeng, D., Wan, J., Li, J., and Zhou, Y. (2010). The rice dynamin-related protein DRP2B mediates membrane trafficking, and thereby plays a critical role in secondary cell wall cellulose biosynthesis. *The Plant Journal* 64**,** 56-70. doi: <https://doi.org/10.1111/j.1365-313X.2010.04308.x>.

Yao, Y., Zhou, J., Cheng, C., Niu, F., Zhang, A., Sun, B., Tu, R., Wan, J., Li, Y., Huang, Y., Xie, K., Dai, Y., Zhang, H., Hong, J.H., Pan, X., Zhu, J., Zhou, H., Liu, Z., Cao, L., and Chu, H. (2022). A conserved clathrin-coated vesicle component, OsSCYL2, regulates plant innate immunity in rice. *Plant, Cell & Environment* 45**,** 542-555. doi: <https://doi.org/10.1111/pce.14240>.

Zhang, H., Zhou, J.-F., Kan, Y., Shan, J.-X., Ye, W.-W., Dong, N.-Q., Guo, T., Xiang, Y.-H., Yang, Y.-B., Li, Y.-C., Zhao, H.-Y., Yu, H.-X., Lu, Z.-Q., Guo, S.-Q., Lei, J.-J., Liao, B., Mu, X.-R., Cao, Y.-J., Yu, J.-J., Lin, Y., and Lin, H.-X. (2022). A genetic module at one locus in rice protects chloroplasts to enhance thermotolerance. *Science* 376**,** 1293-1300. doi: doi:10.1126/science.abo5721.

Zhang, X.-Q., Hou, P., Zhu, H.-T., Li, G.-D., Liu, X.-G., and Xie, X.-M. (2013). Knockout of the VPS22 component of the ESCRT-II complex in rice (*Oryza sativa L*.) causes chalky endosperm and early seedling lethality. *Molecular Biology Reports* 40**,** 3475-3481. doi: 10.1007/s11033-012-2422-1.

Zhou, L., Chen, S., Cai, M., Cui, S., Ren, Y., Zhang, X., Liu, T., Zhou, C., Jin, X., Zhang, L., Wu, M., Zhang, S., Cheng, Z., Zhang, X., Lei, C., Lin, Q., Guo, X., Wang, J., Zhao, Z., Jiang, L., Zhu, S., and Wan, J. (2023). ESCRT-III component OsSNF7.2 modulates leaf rolling by trafficking and endosomal degradation of auxin biosynthetic enzyme OsYUC8 in rice. *Journal of Integrative Plant Biology* 00**,** 1-16. doi: <https://doi.org/10.1111/jipb.13460>.

Zhu, J., Ren, Y., Wang, Y., Liu, F., Teng, X., Zhang, Y., Duan, E., Wu, M., Zhong, M., Hao, Y., Zhu, X., Lei, J., Wang, Y., Yu, Y., Pan, T., Bao, Y., Wang, Y., and Wan, J. (2019). OsNHX5-mediated pH homeostasis is required for post-Golgi trafficking of seed storage proteins in rice endosperm cells. *BMC Plant Biology* 19**,** 295. doi: 10.1186/s12870-019-1911-y.

Zhu, J., Ren, Y., Zhang, Y., Yang, J., Duan, E., Wang, Y., Liu, F., Wu, M., Pan, T., Wang, Y., Hu, T., Hao, Y., Teng, X., Zhu, X., Lei, J., Jing, R., Yu, Y., Sun, Y., Bao, X., Bao, Y., Wang, Y., and Wan, J. (2021). Subunit E isoform 1 of vacuolar H+-ATPase OsVHA enables post-Golgi trafficking of rice seed storage proteins. *Plant Physiology* 187**,** 2192-2208. doi: 10.1093/plphys/kiab099.

Zhu, X., and Chen, X. (2023). A “protector” model for membrane trafficking-regulated and NLR-mediated plant immunity. *Molecular Plant* 16**,** 303-305. doi: <https://doi.org/10.1016/j.molp.2022.12.003>.

Zhu, X., Yin, J., Liang, S., Liang, R., Zhou, X., Chen, Z., Zhao, W., Wang, J., Li, W., He, M., Yuan, C., Miyamoto, K., Ma, B., Wang, J., Qin, P., Chen, W., Wang, Y., Wang, W., Wu, X., Yamane, H., Zhu, L., Li, S., and Chen, X. (2016). The multivesicular bodies (MVBs)-localized AAA ATPase LRD6-6 inhibits immunity and cell death likely through regulating MVBs-mediated vesicular trafficking in rice. *PLOS Genetics* 12**,** e1006311. doi: 10.1371/journal.pgen.1006311.
